# Items, Notices, Etc.

**Published:** 1864-01

**Authors:** 


GRAHAM BREAD.—Take the unbolted flour of wheat, wet it with lukewarm water, add salt and yeast, knead in enough more of this flour to make it stiff, add a little molasses, and, when risen, bake in medium-sized loaves.

ERYSIPELAS is said to be cured by applying to the part affected, a paste made of raw cranberries beaten.

KETTLES are cleansed of onion and other odors, by dissolving a teaspoonful of pearlash or saleratus in water, and washing them.

HAIR, removed by fevers and other sickness, is made to grow by washing the scalp with a strong decoction of sage leaves once or twice a day.

STINGS and bites are often instantaneously cured by washing them in hartshorn or turpentine.

FLIES DESTROYED.—A pint of sweet milk, a quarter of a pound of sugar, two ounces of ground pepper, simmer together for ten minutes, and place it about in shallow dishes. If this is true, there is no necessity for using poisonous articles about a house.

BOILING POTATOES.—It is said that in Ireland they always nick off a piece of the skin, put them in a pot of cold water, which is gradually heated, but never allowed to boil; cold water should be added as soon as the water begins to boil; when done, pour all the water off, cover the vessel with a cloth, and in a few minutes they are cool enough for use.

BUY the best articles for family use; for, although they cost more, good articles spend best.

SUGAR from Havana is always dirty, that from Brazil is clean, as also from Porto Rico and Santa Cruz. Refined sugars, whether loaf, crushed, or granulated, are the cheapest in the end.

LARD from a hog not over a year old, is the best, and should be hard and white.

BUTTER made in September and October is the best for winter use.

RICH cheese feels softer under the pressure of the finger. That which is very strong is neither very good nor healthy. To keep one that is cut, tie it up in a bag that will not admit flies, and hang it in a dry cool place. If mold appears on it, wipe it off with a dry cloth.

FLOUR and meal of all kinds should be kept in a cool dry place.

THE best rice is large, and has a clear fresh look. Old rice sometimes has little black insects inside the kernels.

THE small white sago, called the pearl sago, is the best. The large brown kind has an earthy taste. This article and tapioca, ground rice, etc., should be kept covered.

To select nutmegs, pick them with a pin. If they are good, the oil will instantly spread around the puncture.

KEEP coffee by itself, as the odor affects other articles. Keep tea in a close chest or canister.

ORANGES and lemons keep best wrapped close in soft paper, and laid in a drawer of linen.

THE cracked cocoa is best; but that which is put up in pound papers is often very good.

SOFT soap should be kept in a dry place in the cellar, and not be used until three months old.

To thaw frozen potatoes, put them in hot water. Frozen apples in cold water, but use them at once.

OVER-EATING.—As soon as you are sensible that you have eaten too much, take a walk, gradually increasing its rapidity until there is a free perspiration, and continue at this gait until every feeling of discomfort about the stomach or lungs has disappeared, then cool off very slowly in a closed room, and eat not an atom until the second meal thereafter, thus omitting one.

SICK headache is almost always attended with cold feet, and the failure of a daily action of the bowels; and there is no permanent cure without the rectification of these.

WHITEWASH.—White fences and outbuildings indicate the thrifty farmer and a tidy household. Put half a bushel of unslacked lime in a clean, tight barrel, pour over it boiling water until it is covered five inches, stir briskly until the lime is thoroughly slacked, then add more water until it is as thin as desired, next add two pounds of sulphate of zinc and one of common salt; then apply with a common whitewash brush, giving a good coat in April and October, or at least once a year.

If you get your feet or body wet, keep moving with sufficient briskness to keep off a feeling of chilliness until you get to the house; undress instantly by a warm fire, drinking, as soon as possible, a cup or two of hot tea of any sort, and remain by the fire until thoroughly rested.

When from any cause the bowels fail to act at the usual time, do not eat an atom more until they do act, at least for thirty-six hours; the first meal after a fast should be very light, of bread and butter, and a cup of weak tea or coffee.

Biliousness is indicated by a bad taste in the mouth of mornings, a poor appetite, and a feeling of general discomfort, often accompanied with a headache and cold feet. The best cure is to work moderately, take but two meals a day, and these of bread and butter, with a cup of tea or coffee.

Poison of almost any kind swallowed will be instantly thrown from the stomach by drinking half a glass of water, (warm is best,) in which has been stirred a tablespoon of ground mustard; as soon as vomiting ceases, drink a cup of strong coffee, into which has been stirred the white of an egg; this nullifies any remnant which the mustard might have left.

Paste may be made with flour in the usual way, but rather thicker, with a proportion of brown sugar, and a small quantity of corrosive sublimate. A drop or two of the essential oil of lavender, peppermint, anise, or bergamot, is a complete security against molding. Paste made in this manner, if kept in a close covered pot, may be preserved in a state fit for use at any time.

Liquid glue is made by dissolving a pound of common glue with heat in a pound of strong vinegar, and one quarter of a pound of alcohol; this is whitened by adding sulphate of lead.

Moths are kept from carpets by sprinkling salt and pepper, mixed in equal quantities, about and under the edges.

Bed-bugs are kept away by washing the crevices with strong salt water, put on with a brush.

Picture-frames and glasses are preserved from flies by painting them with a brush dipped in a mixture made by boiling three or four onions in a pint of water.

An ink-stand was turned over on a white table-cloth, a servant threw over it a mixture of salt and pepper plentifully, and all traces of it disappeared.

ANTS are kept out of drawers and other places by spirits of camphor.

BUTTER KEPT FRESH.—Take it as it comes from the churn, and wash the butter-milk thoroughly out of it, then dry the surface of the butter with a clean cloth, break into small pieces, and pack it solid into a crock. The air must be entirely expelled. Set the crock in a kettle half-filled with water, then place the kettle over the fire until the water boils. While boiling remove from the fire, and let the crock remain in the water until cold.

A MEDICINE.—Abernethy's prescription to a wealthy patient was: "Let your servant bring you three or four pails of water, and put it into a wash-tub; take off your clothes, get into it, and from head to foot rub yourself well with it, and you'll recover."

"This advice of yours seems very much like telling me to wash myself," said the patient.

"Well," said Abernethy, "it is open to that objection."

A PRESCRIPTION.—If you wake up thirsty, diet, that is, eat nothing; if you have diarrhea, be quiet, that is, do nothing, drink nothing; and if not better in twelve hours, send for a physician.

LIGHTNING-RODS, in cities, says Prof. Henry, of the Smithsonian Institute, should be connected with the water or gas-pipes underground, outside the building.

A BIT of glue dissolved in skim-milk and water, will restore old crape. Half a cranberry bound on a corn will soon kill it.

RED ANTS.—Wash your shelves down clean, and while damp rub fine salt on them quite thick, and let it remain on for a time, and they will disappear.

EYES.—If you must sew on black cloth at night, pin a piece of soft white paper along the seam, and sew through it; afterwards tear the paper away.

CAMOMILE.—The decoction of its leaves is said to destroy various insects; the living plant imparts health to other plants, often reviving drooping ones.

POTATOES contain nearly all their nutriment (the starch) very near the surface; the heart has but little; hence, let the peeling be the thinnest possible.

LAC stick dissolved in alcohol is the best varnish for tree or vine wounds, and to prevent bleeding from trimming or pruning.

GREENWOOD Cemetery, up to June, 1860, had received seventy-six thousand seven hundred and ninety-seven dead bodies (76,797) since first opened, September 5th, 1840.

The New Reservoir, in the north-eastern portion of the Central Park, is a well whose mouth equals a surface of one hundred and six acres, a well whose depth is twenty feet, and will contain water enough to supply the city of New-York for one month, in case of an accident cutting off the supply from Croton river.

MUSIC FOR OUR DAUGHTERS.—Strangers who are now flocking to the city, will find it to their interest to call at Worcester's piano establishment, corner of Fourteenth street and Third avenue. This is one of the very oldest houses of New-York; in all the financial "crises" of the country, it has never known a "suspension" or a "removal;" thus indicating a thrift, which is only known to men who always make the best instruments, and thus secure the steady patronage of wealthy families, which, from its extent, enables the proprietor to sell a better piano at a lower price than can elsewhere be had, being more especially adapted to withstand the effects of a warm and moist climate.

*How to Enjoy Life.* By Dr. W. M. Cornell. Published by James Challen & Son, Philadelphia, and by Sheldon & Co., New-York. This book contains a large amount of useful truth, and we trust its industrious author will find for it a large sale.

*Movement Cure or Motor-Pathy*, by Dr. George H. Taylor, published by Fowler & Wells, contains a large amount of curious and useful information as to the preservation of health and the removal of disease by muscular and mechanical means.

EYES, CATARACT.—Mark Stephenson, M.D., Surgeon of the New-York Ophthalmic Hospital, etc., read a paper before the American Medical Association convened at Washington City, whose publication was considered of such importance as to be called for by the Society. It reflects great credit on the learned author, for its professional ability, and extensive research.

### HEALTH TRACT, No. 63.

### SKATING

Is one of the most exhilarating of all pastimes, whether on the ice, or over our parlor or hall floors, with roller-skates. In the days of "Queen Bess," some three hundred years ago, it was a favorite amusement with the Londoners, whose facilities for the same were limited to pieces of bone attached to the shoes. As lives have been lost in connection with skating, the following suggestions are made :

1. Avoid skates which are strapped on the feet, as they prevent the circulation, and the foot becomes frozen before the skater is aware of it, because the tight strapping benumbs the foot and deprives it of feeling. A young lady at Boston lost a foot in this way; another in New-York, her life, by endeavoring to thaw her feet in warm water, after taking off her skates. The safest kind are those which receive the fore-part of the foot in a kind of toe, and stout leather around the heel, buckling in front of the ankle only, thus keeping the heel in place without spikes or screws, and aiding greatly in supporting the ankle.

2. It is not the object so much to skate fast, as to skate gracefully ; and this is sooner and more easily learned by skating with deliberation ; while it prevents overheating, and diminishes the chances of taking cold by cooling off too soon afterward.

3. If the wind is blowing, a vail should be worn over the face, at least of ladies and children ; otherwise, fatal inflammation of the lungs, "pneumonia," may take place.

4. Do not sit down to rest a single half-minute ; nor stand still, if there is any wind ; nor stop a moment after the skates are taken off ; but walk about, so as to restore the circulation about the feet and toes, and to prevent being chilled.

5. It is safer to walk home than to ride ; the latter is almost certain to give a cold.

6. Never carry any thing in the mouth while skating, nor any hard substance in the hand ; nor throw any thing on the ice ; none but a careless, reckless ignoramus, would thus endanger a *fellow*-skater a *fall*.

7. If the thermometer is below thirty, and the wind is blowing, no lady or child should be skating.

8. Always keep your eyes about you, looking ahead and upward, not on the ice, that you may not run against some lady, child, or learner.

9. Arrange to have an extra garment, thick and heavy, to throw over your shoulders, the moment you cease skating, and then walk home, or at least half a mile, with your mouth closed, so that the lungs may not be quickly chilled, by the cold air dashing upon them, through the open mouth ; if it passes through the nose and head, it is warmed before it gets to the lungs.

10. It would be a safe rule for no child or lady to be on skates longer than an hour at a time.

11. The grace, exercise, and healthfulness of skating on the ice, can be had, without any of its dangers, by the use of skates with rollers attached, on common floors ; better if covered with oil-cloth. Lessons are given in this pleasant and exhilarating exercise at Mr. Disbrow's on Fifth Avenue, whose spacious and well-conducted establishment ought to be well patronized. His ice pond is now in exellent order.

### NOTICES.

ATLANTIC MONTHLY, published by Ticknor & Fields, No. 135 Washington street, Boston, Mass.; three dollars a year; Volume 13 began with January. Among its contributors are some of the best intellects and the most cultivated minds in the nation. It is the great literary monthly of the country, and by the acknowledged ability with which it has been conducted, it has been placed on a permanent basis, and is highly appreciated abroad as well as at home.

THE HORTICULTURIST, founded by the lamented A. J. Downing, in 1846, is in its eighteenth volume: two dollars a year; No. 37 Park Row, New-York. Its patronage is commended to country gentlemen and intelligent agriculturists throughout the country.

THE PRESBYTERIAN, Philadelphia, two dollars and fifty cents a year, has an industrious and able correspondent in this city; his weekly letters well pay for the subscription-price to every New-Yorker who wishes to keep himself posted as to the "goings on" and chief doings of our mighty metropolis.

TO FARMERS.—There is no monthly published on this or any other continent on agriculture or on any other subject which gives one half as much valuable, practical, and reliable information for one dollar a year as the *American Agriculturist*, issued at No. 7 Park Row, New-York City.

BLACKWOOD'S MAGAZINE, two dollars a year, *London Quarterly*, *The Edinburgh*, *The Westminster*, and *North British Reviews*, each three dollars, are all furnished for ten dollars a year. Address Leonard Scott & Co., No. 54 Gold street, New-York. The contributors to these publications are among the very ablest writers in Great Britain.

MUSIC.—The hinged-plate piano improvement of Horatio Wooster, of New-York, is eliciting the admiration and hearty commendation of the most accomplished artists in the country. Among the names are those of Gottschalk, Muzio, Mason, Berge, Fredel, Thomas, Harrison, Wernike, Morgan, Goschö, and the distinguished amateur Dr. Thomas Ward, all substantiating the sentiment of Gottschalk, when he said, "I estimate the volume of tone to be increased one hundred per cent by this invention," which is certainly very high praise, coming as it does from the very highest musical authority.

GODEY'S LADY'S BOOK, three dollars a year, Philadelphia, continues as heretofore to be the Queen of pictorial monthlies, delighting multitudes of families with its beautiful steel engravings and its valuable practical embellishments, etc.

ARTHUR'S HOME MAGAZINE, two dollars a year, Philadelphia. Who that has ever subscribed for it, ever willingly failed to "renew" when Christmas came?

PILES, FISTULA, RUPTURES, ETC.—The last published work of Dr. Bodenhemer on these and kindred subjects has been translated into French. Dr. B. spends the winter at the Monongahela House, Pittsburgh, and for knowledge, ability, skill, and success has no superior living.

TEETH.—Dr. John Allen, 22 Bond street, New-York, in whose office Dr. Evans, now the first Dentist in Europe, took lessons is believed to be the ablest member of his profession for furnishing single and sets of teeth. We know cases where twenty years of youthfulness have been imparted to the features.

THE Farm-House Milk, pure and sweet, is brought to town daily by the New-Jersey and Rockland County Milk Association, under the management of C. W. Canfield, Esq., No. 146 Tenth street, New-York, near Broadway, adjoining Stewart's New Retail Palace.

BARNUM'S MUSEUM continues to be the general place of resort for novelty-seekers. Formerly a "Museum" was considered to be a collection of all the queer, outlandish things of creation, but Mr. Barnum, with characteristic energy and forecast, has made his establishment a place not only of amusement but of solid instruction. Scarcely a week passes in which some new object of interest is not introduced. Natural treasures are gathered from the poles to the tropics; yesterday he had a polar bear; to-day a family of Esquimaux; to-morrow it will be a whale, or a multitude of fishes of all sizes and hues, from the Pole to the Line; and frequently all are seen at once, exciting the mind of the beholder alternately with feelings of awe, of wonder, of admiration and delight.

IRON FENCES, railings, plain and ornamental, statues, figures of animals, bedsteads, gate-posts, tree-guards, with every conceivable variety of article for families, farms, cemeteries, parks and pleasure-gardens and grounds, are found at the very extensive establishment of Hutchinson & Wickersham, 259 Canal street, New-York, one of the oldest and best known houses of the kind in the city.

WE heartily commend "The Home Monthly," two dollars a year, Boston, to every household wishing a whole year of delightful and instructive reading for wives, husbands, daughters, and sons.

